# Loss of GAS5 tumour suppressor lncRNA: an independent molecular cancer biomarker for short-term relapse and progression in bladder cancer patients

**DOI:** 10.1038/s41416-018-0320-6

**Published:** 2018-10-30

**Authors:** Margaritis Avgeris, Anastasia Tsilimantou, Panagiotis K. Levis, Theodoros Tokas, Diamantis C. Sideris, Konstantinos Stravodimos, Alexandros Ardavanis, Andreas Scorilas

**Affiliations:** 1 Department of Biochemistry and Molecular Biology, Faculty of Biology, National and Kapodostrian University of Athens, Athens, Greece; 2 First Department of Urology, “Laiko” General Hospital, Medical School, National and Kapodostrian University of Athens, Athens, Greece; 3 First Medical Oncology Clinic, “Saint Savvas” Anticancer Hospital, Athens, Greece

**Keywords:** Bladder cancer, Translational research, Tumour biomarkers, Prognostic markers, Molecular medicine

## Abstract

**Background:**

Bladder cancer (BlCa) heterogeneity and the lack of personalised prognosis lead to patients’ highly variable treatment outcomes. Here, we have analysed the utility of the GAS5 tumour-suppressor lncRNA in improving BlCa prognosis.

**Methods:**

GAS5 was quantified in a screening cohort of 176 patients. Hedegaard et al. (2016) (*n* = 476) and TCGA provisional (*n* = 413) were used as validation cohorts. Survival analysis was performed using recurrence and progression for NMIBC, or death for MIBC. Internal validation was performed by bootstrap analysis, and decision curve analysis was used to evaluate the clinical benefit on disease prognosis.

**Results:**

GAS5 levels were significantly downregulated in BlCa and associated with invasive high-grade tumours, and high EORTC-risk NMIBC patients. GAS5 loss was strongly and independently correlated with higher risk for NMIBC early relapse (HR = 2.680, *p* = 0.011) and progression (HR = 6.362, *p* = 0.035). Hedegaard et al. and TCGA validation cohorts’ analysis clearly confirmed the association of GAS5 loss with NMIBC worse prognosis. Finally, multivariate models incorporating GAS5 with disease established markers resulted in higher clinical benefit for NMIBC prognosis.

**Conclusions:**

GAS5 loss is associated with adverse outcome of NMIBC and results in improved positive prediction of NMIBC patients at higher risk for short-term relapse and progression, supporting personalised prognosis and treatment decisions.

## Background

Bladder cancer (BlCa) represents the second most common urologic cancer and the fourth most commonly diagnosed malignancy among the male population in developed countries,^1,2^ with the majority of the tumours (90%) originating in the bladder urothelium. Urothelial bladder carcinoma is a disease spectrum classified into two main clinical groups, depending on the invasion into the detrusor muscle. Approximately 75% of urothelial bladder carcinomas represent non-muscle-invasive bladder cancer (NMIBC) (Ta, Tis, T1), not invading the detrusor muscle, while the other 25% accounts for muscle-invasive bladder cancer (MIBC) (T2–T4).^3^

Disease-specific mortality has been significantly reduced nowadays, due to smoking reduction and to outstanding improvements in disease diagnosis and management—based on evolution of imaging, new diagnostic modalities and advanced surgical techniques.^1,4^ Despite however the administration of active treatment, BlCa frequently recurs and become life threatening.^5^ NMIBC, although not associated with mortality per se, is characterised by strong recurrence potential and subsequently by progression to muscle-invasive tumours, which are metastatic and lethal. In the clinical setting, disease prognosis relies on tumour stage and grade, as well as on disease multifocality, tumour size and concomitant carcinoma in situ (CIS).^6,7^ In this regard, EORTC-risk-group stratification represents the clinically established and used predictor of NMIBC outcome.^8,9^ However, tumours’ heterogeneity in the cellular and molecular levels is responsible for the highly variable clinical outcome of the same risk-group patients, making disease prognosis a rather demanding task. Consequently, the elucidation of disease molecular hallmarks could effectively improve patients’ prognostication and support precision medicine.

The growth arrest-specific 5 (GAS5) long non-coding RNA (lncRNA; ~0.7 kb), originally identified by Schneider et al. through cDNA cloning of genes overexpressed in growth-arrested cells, is encoded by the intergenic *GAS5* gene at the region 1q25.1.^10^ *GAS5* is comprised of 12 exons, bearing a 5′-terminal oligopyrimidine (5′-TOP) sequence in exon 1, and beside several mature GAS5 variants, *GAS5* encodes 10 C/D box snoRNAs (SNORD44, SNORD47, SNORD74–SNORD81) within its 11 introns.^11^ Although a short open reading frame, able to encode for a 50 amino acid polypeptide, has been identified within gene exons, *GAS5* sequence is poorly conserved and does not encode any functional protein.^11,12^ *GAS5* transcription is regulated by the interplay of mTOR and nonsense-mediated decay pathways, resulting in the increased GAS5 levels in growth-arrested cells.^13–15^

The well-characterised tumour suppressor of GAS5 has been documented in a wide variety of human malignancies and loss of GAS5 expression has been implicated both in tumorigenesis and disease progression,^16,17^ as well as in patients prognosis.^18,19^ A dual complementary function in arresting cell growth by inhibition of cell proliferation and stimulation of apoptosis has been attributed to GAS5, highlighting an active role in growth arrest rather than a passive association. Indeed, GAS5 silencing is associated with increased proportion of cells in the S/G2 phase as well as with attenuated apoptosis upon endogenous stimuli or chemotherapeutic agents.^20–22^ The main molecular mechanisms of GAS5 tumour-suppressor activity are riborepression of glucocorticoid receptor (GR) transcriptional activity and “sponging” of oncogenic miRNAs.^16,17,23^

Despite the crucial tumour-suppressive role of GAS5 lncRNA in the molecular background of BlCa establishment and progression,^24–27^ there is no complete evaluation of its clinical utility for the patients. In the present study, for the first time, we have evaluated the clinical value of GAS5 tumour-suppressor lncRNA in improving patients’ prognosis and prediction of disease course.

## Patients and methods

### Screening cohort

The biological samples analysed in the study consisted of 363 fresh-frozen bladder tissue specimens. Bladder tumours obtained from 176 BlCa patients who underwent transurethral resection of bladder tumours (TURBT) for primary NMIBC or radical cystectomy (RC) for primary MIBC at the “Laiko” General Hospital, Athens, Greece, representing our screening patients’ cohort. Adjacent normal bladder tissues were also obtained from 144 patients of the screening cohort, following pathologist’s evaluation for the absence of dysplasia or CIS. Prior to surgery none of the patients received any form of neoadjuvant treatment. Finally, healthy bladder tissue samples were available from 43 benign prostate hyperplasia patients who underwent transvesical suprapubic prostatectomy. Tissue specimens were incubated in RNAlater Solution (Ambion, Carlsbad, CA, USA) according to the manufacturer’s instructions and stored −80 °C until analysis.

NMIBC patients received adjuvant therapy according to European Association of Urology (EAU) guidelines, while MIBC patients did not receive any form of adjuvant treatment. NMIBC patients’ risk-group stratification was performed according to the European Organization for Research and Treatment of Cancer (EORTC) guidelines. NMIBC patients were followed-up by cystoscopy and urinary cytology (for high-grade (HG) tumours) according to EAU guidelines. MIBC patients’ follow-up included renal ultrasound at 3 months and thoracoabdominal computed tomography (CT)/magnetic resonance imaging (MRI) every 6 months, while additional kidney ultrasound and thoracoabdominal CT/MRI, as well as bone scan or brain MRI was only performed following symptoms. Disease recurrence (the same or lower stage) and progression (recurrence of higher/invasive stage) of NMIBC patients were confirmed by histology findings of a TURBT that performed after a positive follow-up cystoscopy, while in MIBC patients’ disease recurrence was detected by a follow-up CT.

The present study was conducted according to ethical standards of the 1975 Declaration of Helsinki, as revised in 2008, and approved by the ethics committee of “Laiko” General Hospital. Informed consent was obtained by all the participated patients.

### Validation cohorts

The cohorts of Hedegaard et al. (*n* = 476)^28^ and TCGA (The Cancer Genome Atlas, provisional) (*n* = 413)^29^ were used as our study validation cohorts regarding NMIBC and MIBC, respectively. Hedegaard et al. performed paired-end whole transcriptome, strand-specific RNA-seq (Illumina HiSeq platform) of a cohort consisted of 460 NMIBC (Ta: 345, T1: 112, CIS: 3) and 16 MIBC patients. TCGA (provisional) cohort (*n* = 413) consisted of 409 MIBC patients, and lncRNA expression profiles generated by paired-end whole transcriptome RNA-seq (Illumina HiSeq platform). Clinical and normalised expression data were downloaded for Hedegaard et al. cohort by EMBL-EBI ArrayExpress (accession number ArrayExpress: E-MTAB-4321; https://www.ebi.ac.uk/arrayexpress/experiments/E-MTAB-4321/) and for TCGA cohort (provisional) by cbioportal (http://www.cbioportal.org/).^30^

### Extraction of total RNA

Following pulverisation of 40–150 mg of fresh-frozen tissue specimen, total RNA was extracted using TRI-Reagent (Molecular Research Center, Cincinnati, OH, USA) according to the manufacturer’s instructions, dissolved in RNA Storage Solution (Ambion) and stored at −80 °C. RNA concentration and purity were determined spectrophotometrically at 260 and 280 nm, while agarose gel electrophoresis was performed to evaluate RNA integrity.

### First-strand cDNA synthesis

Total RNA was reverse transcribed in a 20 μl reaction containing 1 μg of total RNA template, 50 U MMLV reverse transcriptase (Invitrogen, Carlsbad, CA, USA), 40 U recombinant ribonuclease inhibitor (Invitrogen) and 5 μM oligo-dT primers. Reverse transcription took place at 37 °C for 60 min, while enzyme inactivation performed at 70 °C for 15 min.

### Quantitative real-time PCR

A SYBR-Green fluorescent-based quantitative real-time PCR (qPCR) assays was developed and applied to assess GAS5 expression. Based on published sequences (NCBI Ref Seq: NR_002578.3 for *GAS5* and NM_000194.2 for *HPRT1*) and according to in silico analysis, specific primers for *GAS5* (F: 5′-CTTGCCTGGACCAGCTTAAT-3′, R: 5′-CAAGCCGACTCTCCATACCT-3′) and *HPRT1* (F: 5′-TGGAAAGGGTGTTTATTCCTCAT, R: 5′-ATGTAATCCAGCAGGTCAGCAA-3′) were designed and used for the amplification of a 122 bp *GAS5*-specific and a 151 bp *HPRT1*-specific amplicon.

The 7500 Real-Time PCR System (Applied Biosystems, Carlsbad, CA) was used for the qPCR assays. The 10 μl reaction consists of Kapa SYBR^®^ Fast Universal 2X qPCR MasterMix (Kapa Biosystems, Inc., Woburn, MA), 100 nM of each specific PCR primer, and 10 ng of cDNA template. The thermal protocol consisted of polymerase activation step at 95 °C for 3 min, followed by 40 cycles of denaturation at 95 °C for 15 s and finally the primer annealing and extension step at 60 °C for 1 min. Melting curve analysis and agarose gel electrophoresis were performed following amplification to discriminate specific amplicons from non-specific products or primer dimers.

The ${2}^{{{-\Delta\Delta}{\mathrm{C}}}_{\mathrm{T}}}$ relative quantification (RQ) method was used to quantify GAS5 levels. Duplicate reactions were performed for each tested sample and target, and the average C_T_ was calculated and used for the quantification analysis. RT112 bladder cancer cell line was used as a calibrator and *HPRT1* as endogenous reference control for normalisation purposes.

### Statistical analysis

The IBM SPSS Statistics 20 software (IBM Corp., Armonk, New York, USA) was used for the statistical analysis. Non-parametric tests were applied appropriately in order to analyse GAS5 levels differences between tumours and healthy bladder urothelium, as well as to assess the correlation of GAS5 expression with patients’ clinicopathological features. The ability of GAS5 to discriminate bladder tumours from normal urothelium was evaluated by the ROC curve and logistic regression analysis.

Patients’ survival analysis performed by Kaplan–Meier survival curves and Cox proportional regression analysis. Internal validation was performed by bootstrap Cox proportional regression analysis based on 1000 bootstrap samples. Optimal cut-off values of the GAS5 expression levels in NMIBC and MIBC patients’ cohorts were determined using the X-tile algorithm. To evaluate the clinical net benefit of the prediction models on disease outcome following treatment, decision curve analysis was performed according to Vickers et al.,^31^ using the STATA 13 software (StataCorp LLC, College Station, TX, USA).

## Results

### Baseline clinical and experimental data

**Fig. 1:**
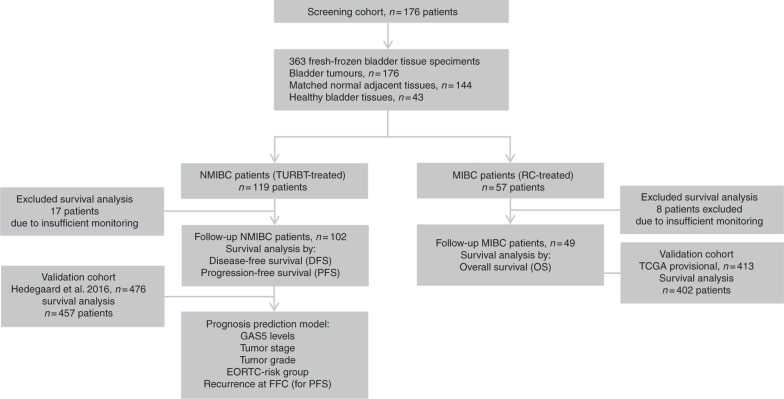
REMARK diagram of the study

The REMARK diagram of our study is presented in Fig. 1. Patients’ screening cohort consisted mostly of males (83.5%) with a median age of 70 years. Concerning disease pathology, 67.6% and 32.4% of the patients were diagnosed and treated for primary NMIBC (TaT1) and MIBC (T2–T4), respectively. Within the T1 cohort, 34.5% and 65.5% of the tumours were of low-grade (LG) and HG, respectively, while the vast majority of the MIBC patients (96.5%) displayed HG tumours. According to EORTC-risk-stratification guidelines, 12.6%, 31.9% and 55.5% of the enrolled NMIBC patients were classified as low, intermediate and high risk, respectively. BlCa patients’ clinicopathological characteristics are presented in Supplementary Table 1.

Regarding patients’ outcome following treatment, 151 patients were successfully followed-up, whereas 25 patients were excluded due to insufficient and unclear monitoring data. During a median follow-up time (reverse Kaplan–Meier method) of 31 months (95% CI: 29.16–32.84), of the 102 follow-up TaT1 patients, disease recurrence and progression were detected in 40 (39.2%) and 15 (14.7%) patients, respectively, with 17 patients (16.7%) experiencing recurrence at the first follow-up cystoscopy (FFC). Focusing on MIBC, 25 of the 49 follow-up patients died (51.0%). The mean disease-free survival (DFS) and progression-free survival (PFS) of the NMIBC patients was 30.76 (95% CI: 27.24–34.29) and 43.20 months (95% CI: 40.46–45.94), respectively, while the overall survival (OS) of the MIBC patients was 28.11 months (95% CI: 22.91–33.30).

### The expression GAS5 is significantly reduced in bladder tumours

**Fig. 2:**
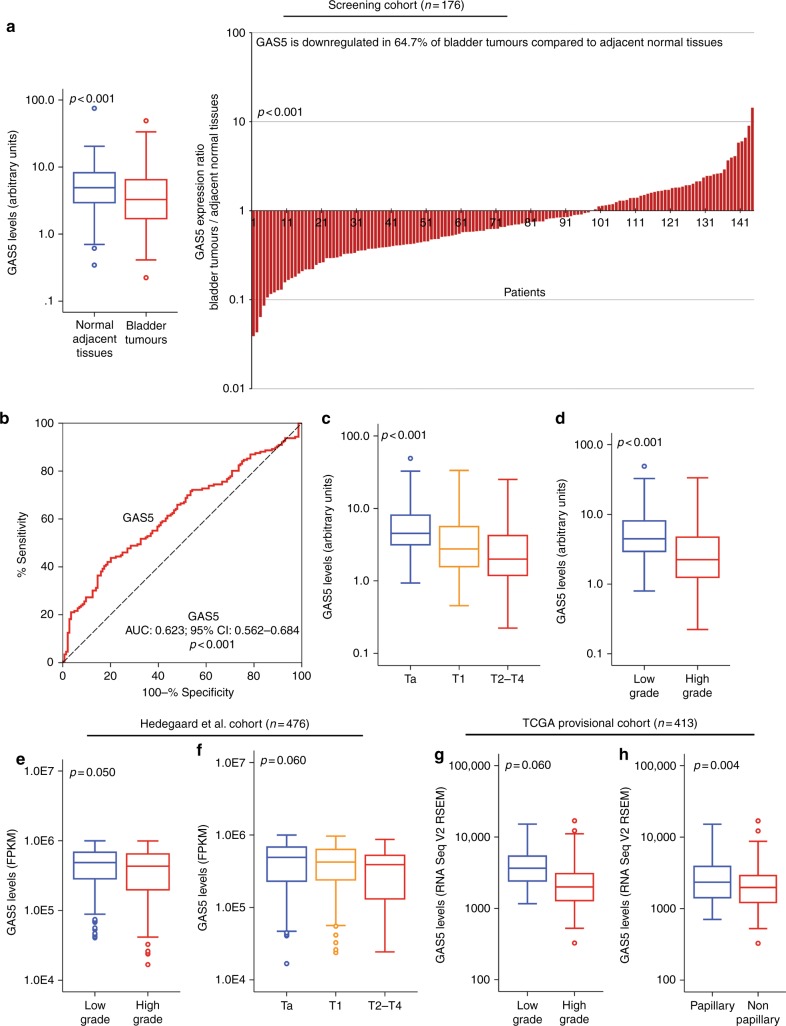
GAS5 expression analysis in BlCa. **a** Box plot and bar graph presenting GAS5 levels and GAS5 levels ratio, respectively, in bladder tumours and matched adjacent normal tissue specimens of bladder cancer patients. *p*-Values calculated by Wilcoxon Singed Rank test. **b** ROC curve analysis of GAS5 levels for the discrimination of bladder tumours patients from matched adjacent normal specimens. *p*-Value calculated by the Hanley and McNeil method. AUC area under the curve, 95% CI 95% confidence interval. **c**, **d** Box plots presenting the correlation of GAS5 levels with tumour stage (**c**) and tumour grade (**d**) in the screening cohort. *p*-Values calculated by Kruskal–Wallis test (**c**) and Mann–Whitney *U*-test (**d**). **e**, **f** Box plots presenting the correlation of GAS5 levels with tumour grade (**e**) and tumour stage (**f**) in the Hedegaard et al. validation cohort. *p*-Values calculated by Mann–Whitney *U*-test (**e**) and Kruskal–Wallis test (**f**). **g**, **h** Box plots presenting the correlation of GAS5 levels with tumour grade (**g**) and tumour growth (histologic subtype) (**f**) in the TCGA provisional validation cohort. *p*-Values calculated by Mann–Whitney *U-*test

The expression analysis (Fig. 2) revealed that GAS5 levels are significantly downregulated (*p* = 0.001) in bladder tumours compared to the matched adjacent normal specimens in 67.4% of the screened patients (Fig. 2a). This finding was confirmed in NMIBC patients, where decreased GAS5 expression (*p* = 0.002) in tumours compared to their matched normal counterparts was detected in approximately 69.5% of the enrolled TaT1 patients (Supplementary Fig. 1). Reduced GAS5 levels in muscle-invasive tumours, while not statistically significant, were also observed in 63.6% of the MIBC (T2–T4) patients (*p* = 0.181; Supplementary Fig. 1). ROC curve analysis highlighted the ability of GAS5 reduced levels to discriminate bladder tumours from the matched normal urothelium (AUC: 0.623; 95% CI: 0.562–0.684; *p* < 0.001; Fig. 2b), which was also confirmed by logistic regression analysis (OR: 0.339; 95% CI: 0.188–0.614; *p* < 0.001; Supplementary Table 2).

### Loss of GAS5 is associated with unfavourable prognostic disease features

The analysis of GAS5 expression regarding patients’ clinicopathological features clearly highlighted the association of GAS5 loss with unfavourable disease prognostic markers (Fig. 2). Significantly lower GAS5 levels (*p* < 0.001) are expressed in muscle-invasive (T2–T4) and connective tissue-invasive (T1) tumours compared to superficial Ta tumours (Fig. 2c), as well as in HG tumours related to LG ones (*p* < 0.001; Fig. 2d). Analysis of the validation cohorts confirmed the reduced expression of GAS5 in HG tumours (Hedegaard et al., *p* = 0.050—PUNLMP not evaluated; Fig. 2e and TCGA, *p* < 0.001; Fig. 2f), and in advanced tumour stages (Hedegaard et al., *p* = 0.060; Fig. 2g). Moreover, the TCGA cohort highlighted the loss of GAS5 levels in non-papillary tumours compared to papillary ones (*p* = 0.004; Fig. 2h).

**Fig. 3:**
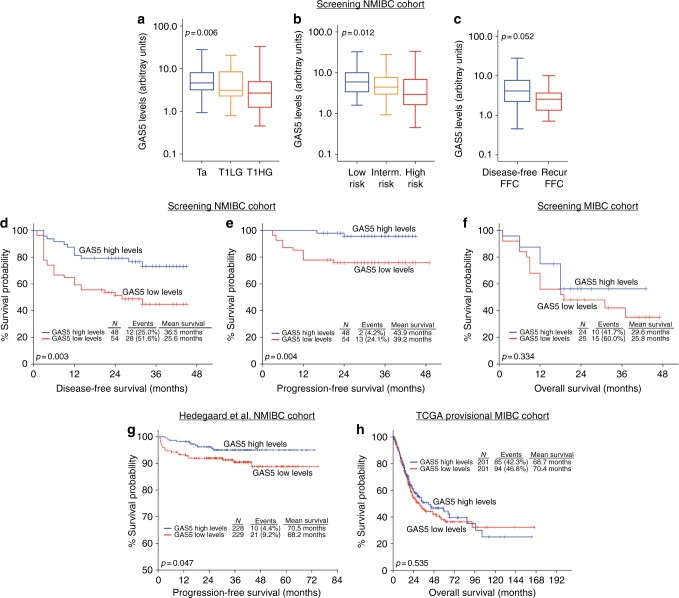
NMIBC (TaT1) patients with reduced GAS5 levels are at significant higher risk for short-term disease recurrence and progression. **a**–**c** Box plots presenting the correlation of GAS5 levels with NMIBC patients’ stage and grade (**a**), EORTC-risk group (**b**) and recurrence at the first follow-up cystoscopy (FFC) (**c**) of the screening cohort. *p*-Values calculated by Kruskal–Wallis test (**a**, **b**) and Mann–Whitney *U-*test (**c**). **d**–**f** Kaplan–Meier survival curves for the disease-free survival (DFS) and progression-free survival (PFS) of the NMIBC (TaT1) patients, as well as the overall survival (OS) of the MIBC (T2–T4) patients according to GAS5 levels in the screening cohort. **g** Kaplan–Meier survival curves for the progression-free survival (PFS) of the NMIBC (TaT1) patients according to GAS5 levels in the Hedegaard et al. validation cohort. **h** Kaplan–Meier survival curves for the overall survival (OS) of the MIBC (T2–T4) patients according to GAS5 levels in the TCGA provisional validation cohort. *p*-Values of the Kaplan–Meier survival analysis calculated by log-rank test

Focusing on NMIBC, significantly downregulated GAS5 levels detected in HG compared to LG TaT1 tumours (*p* = 0.006; not shown) and mainly to T1HG (*p* = 0.006; Fig. 3a). Moreover, the loss of GAS5 was also correlated with higher EORTC-risk group (*p* = 0.012; Fig. 3b) as well as with NMIBC patients suffering disease recurrence at FFC (*p* = 0.052; Fig. 3c).

### Loss of GAS5 is associated with significantly higher risk for recurrence and progression of NMIBC (TaT1) patients

The survival analysis of the screening cohort performed using disease recurrence and disease progression as clinical endpoint events for the DFS and PFS of the NMIBC (TaT1) patients, or patients’ death for the OS of the MIBC (T2–T4) patients (Fig. 3). Using the X-tile algorithm, the 50th percentile (median) of GAS5 levels was adopted as the optimal cut-off value for both NMIBC and MIBC patient groups. Kaplan–Meier curves clearly demonstrated the significantly shorter DFS (*p* = 0.003; Fig. 3d) and PFS (*p* = 0.004; Fig. 3e) expectancy of the TaT1 patients with decreased GAS5 levels compared to the patients overexpressing GAS5. Unfortunately, the analysis of GAS5 tumour levels did not highlight a statistically significant prognostic value for the OS of the MIBC patients (Fig. 3f).

**Fig. 4:**
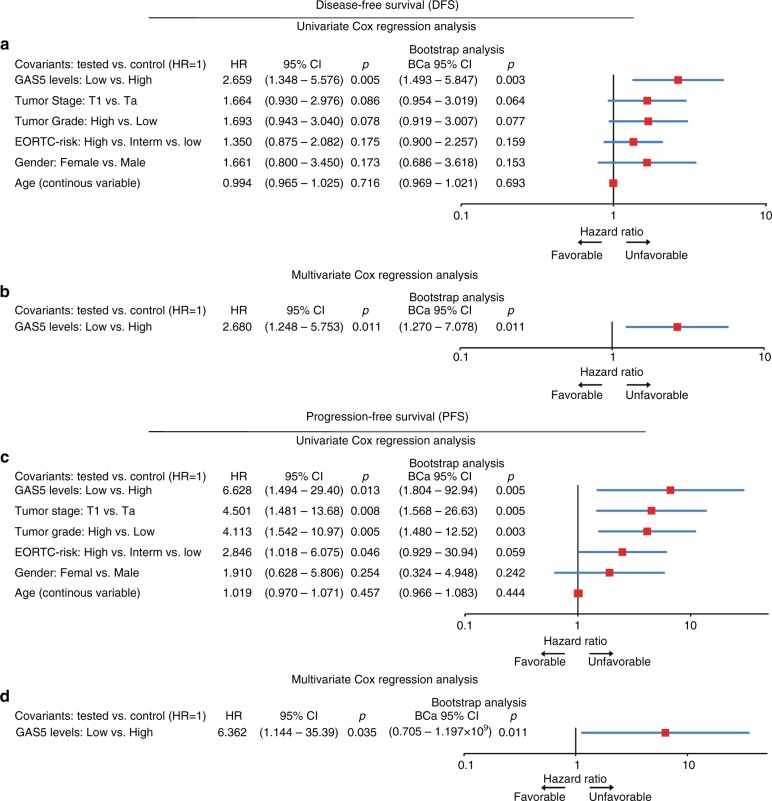
Loss of GAS5 expression represent an independent prognostic predictor of NMIBC (TaT1) patients’ short-term disease recurrence and progression following treatment. Forest plots of the univariate (**a**, **c**) and multivariate (**b**, **d**) Cox regression analysis for the NMIBC (TaT1) patients’ disease-free survival (DFS) and progression-free survival (PFS). Multivariate analysis adjusted for GAS5 expression levels, tumour stage, tumour grade, EORTC-risk stratification, patient gender and age. Internal validation was performed by bootstrap Cox proportional regression analysis based on 1000 bootstrap samples. HR: hazard ratio; 95% CI: 95% confidence interval of the estimated HR; BCa 95% CI: bootstrap bias-corrected and accelerated 95% confidence interval of the estimated HR based on 1000 bootstrap samples

The correlation GAS5 loss with the poor treatment outcome of the NMIBC patients was also confirmed by Cox proportional regression analysis (Fig. 4 and Supplementary Table 3). Univariate analysis highlighted the significantly stronger risk for disease recurrence (HR: 2.659; 95% CI: 1.348–5.246; *p* = 0.005) and progression (HR: 6.628; 95% CI: 1.494–29.40; *p* = 0.013) of the TaT1 patients with downregulated GAS5 levels. Moreover, multivariate Cox models adjusted for tumour stage, grade, EORTC-risk group, patients’ gender and age demonstrated the independent clinical predictive value of GAS5 loss for NMIBC relapse (HR: 2.680; 95% CI: 1.248–5.753; *p* = 0.011) and progression (HR: 6.362; 95% CI: 1.144–35.39; *p* = 0.035).

The analysis of the Hedegaard et al. validation cohort clearly confirmed the unfavourable significance of GAS5 loss for the NMIBC disease course. Using disease progression to muscle-invasive tumours as the clinical endpoint event for the survival outcome of the TaT1 patients (*n* = 457), patients with downregulated GAS5 levels suffered from significantly shorter PFS, as highlighted by Kaplan–Meier curves (*p* = 0.047; Fig. 3g). The survival analysis of the MIBC patients of the TCGA cohort (*n* = 402) did not highlight a statistically significant association of GAS5 levels with patients’ OS, in agreement with the survival analysis of the MIBC patients of our screening cohort (Fig. 3h).

### The evaluation of GAS5 expression improves the clinical value of the established prognostic markers for NMIBC

The powerful and independent prognostic significance for NMIBC prompted us to study GAS5 ability to strengthen the clinical value of the established disease prognostic markers. Tumour stage, grade, EORTC-risk group and recurrence at the FFC (for disease progression) represent the established and widely clinically used prognostic markers for NMIBC, whereas T1HG tumours, high-risk EORTC stratification and recurrence at the FFC (3 months) are independent predictors of TaT1 recurrence and progression following treatment.

**Fig. 5:**
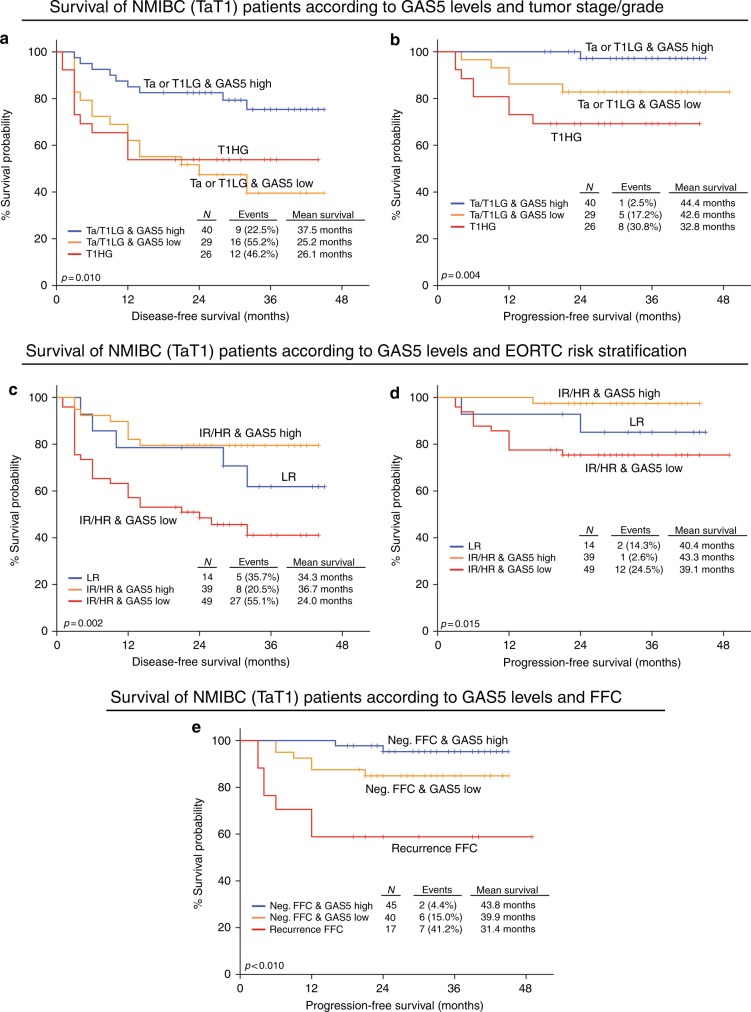
Evaluation of GAS5 levels benefits the prediction of NMIBC (TaT1) patients' risk for disease relapse and progression according to the clinically established prognostic disease markers. Kaplan–Meier survival curves of the NMIBC (TaT1) patients’ disease-free survival (DFS) and progression-free survival (PFS) according to the combination of GAS5 levels with tumour stage and grade (**a**, **b**), EORTC-risk stratification (**c**, **d**), and recurrence at the first follow-up cystoscopy (**e**). *p*-Values calculated by log-rank test

The incorporation of GAS5 levels with the above-mentioned clinically used markers clearly resulted in superior positive prediction of NMIBC patients’ adverse outcome (Fig. 5). Indeed, Ta/T1LG patients with lower GAS5 levels were at significantly higher risk for short-term relapse (*p* = 0.010; Fig. 5a) and progression (*p* = 0.004; Fig. 5b), similar to T1HG patients’ risk. Additionally, GAS5 loss could effectively distinguish patients at higher risk for short-term relapse (*p* = 0.002; Fig. 5c) and progression (*p* = 0.015; Fig. 5d) within the highly heterogeneous cohort of intermediate/high-risk patients according to EORTC-risk stratification. Finally, patients with negative FFC and loss of GAS5 expression presented significantly shorter PFS expectancy (*p* < 0.001; Fig. 5e) compared to those overexpressing GAS5.

### The evaluation of GAS5 levels results in advanced clinical benefit in NMIBC (TaT1) prognosis

**Fig. 6:**
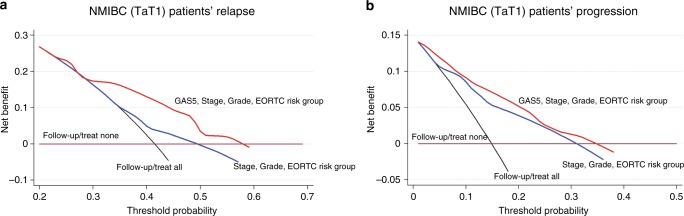
Decision curve analysis highlights the superior clinical net benefit of models including GAS5 loss, compared to models of the clinically established prognostic markers, for the prediction of NMIBC (TaT1) patients’ relapse and progression. Decision curves of prediction models for NMIBC (TaT1) patients’ relapse (**a**) and progression (**b**). Net benefit is plotted against various ranges of threshold probabilities

To evaluate the clinical benefit of the models including GAS5 along with the established disease prognostic markers, decision curve analysis was performed according to Vickers et al. Decision curves of the prediction models tested are presented in Fig. 6. The analysis highlighted the significantly improved clinical benefit of the model incorporating GAS5 loss in predicting NMIBC relapse for threshold probabilities ≥25%, compared to the tumour stage, grade and EORTC-risk group model (Fig. 6a). Similarly, the model integrating GAS5 loss offers the highest clinical benefit for the prediction of NMIBC progression for threshold probabilities >0%, compared to tumour stage, grade, EORTC-risk group and recurrence at FFC model (Fig. 6b). Considering that NMIBC progression to muscle-invasive tumours represents a particularly aggressive clinical event, demanding more intensive follow-up and treatment adjustment, the superior net benefit even at low threshold probabilities is crucial for the effective prognosis and personalised management of the NMIBC patients.

## Discussion

Despite reduction of disease-specific mortality and improvement in disease diagnosis,^1^ urothelial bladder carcinoma remains a clinically heterogeneous malignancy regarding patients’ prognosis and treatment outcome. Indeed, patients of the same risk group and sharing similar clinicopathological features could display highly variable clinical outcomes and responses to therapy.^9^ The lack of accurate disease prognosis forces the generic and non-personalised active treatment and lifelong surveillance of patients, classifying BlCa as the most expensive per-patient-to-treat cancer for the healthcare systems of developed countries.^32^ Consequently, the identification of novel disease markers represents a top clinical priority in order to be able to support personalised treatment and monitoring decisions, to limit unnecessary interventions and healthcare costs, and finally to benefit patients quality-of-life.

The family of non-coding RNAs (ncRNAs) includes a large number of different entities as approximately 70% of the genome is actively transcribed to ncRNAs.^33^ Until recently, miRNAs had received the most attention regarding the role and clinical impact of the family in human malignancies.^34,35^ However, the functional role of lncRNAs in gene expression as well as their deregulated levels in the vast majority of human malignancies resulted in the ever-growing interest in assessing their implication in cancer cell homoeostasis and their clinical impact for the patients.^36^ The aim of the present study was the first-time complete evaluation of GAS5 lncRNA significance in supporting BlCa personalised prognosis and prediction of disease outcome.

The analysis of our screening cohort highlighted the significant reduced expression of GAS5 in bladder tumours compared to their matched adjacent normal urothelium, as well as the ability of GAS5 to discriminate bladder tumours from their normal counterparts. The loss of GAS5 was strongly correlated with unfavourable disease prognostic markers, such as HG carcinomas, as well as muscle-invasive (T2–T4) and T1HG tumours. In this regard, loss of GAS5 expression was significantly associated with high-risk NMIBC according to EORTC stratification and with NMIBC patients displaying recurrence at the FFC.

The survival analysis revealed the independent and unfavourable significance of GAS5 loss for NMIBC patients’ prognosis. Indeed, NMIBC patients underexpressing GAS5 were at significantly higher risk for disease short-term relapse and progression to invasive tumour stages. Additionally, the adverse outcome of the TaT1 patients with GAS5 loss revealed to be independent of tumour stage and grade, EORTC-risk stratification, age and gender. To confirm our findings, Hedegaard et al. (*n* = 476) and TCGA provisional (*n* = 413) bladder urothelial carcinoma cohorts were used as validation cohorts for NMIBC and MIBC, respectively.^28,29^ The analysis of the validation cohorts confirmed the correlation of GAS5 loss with tumours of high grade, higher stage and of non-papillary histology. Moreover, Hedegaard et al. validation cohort for NMIBC clearly verified the significantly worse PFS expectancy of the TaT1 patients underexpressing GAS5. On the other hand, MIBC survival analysis of both the screening cohort and the TCGA validation cohort did not show a statistically significant association with patients’ survival outcome. This observed discrepancy of GAS5 clinical value for NMIBC and MIBC patients could possibly be attributed to the well-documented diversity of non-muscle-invasive (superficial) and muscle-invasive tumours, regarding cellular origin and molecular background,^37–40^ and clearly highlights the NMIBC-specific prognostic utility of GAS5.

Our findings are in line with the well-documented tumour-suppressor role of GAS5, through the inhibition of cell proliferation and the stimulation of apoptosis, in the wide range of human malignancies studied so far.^16,17,41–45^ Repression of GR transcriptional activity^15,23^ and miRNA sponging,^46–50^ have been proposed as the main molecular mechanisms underlying GAS5 tumour-suppressor function. Focusing on BlCa, silencing of GAS5 resulted in enhanced cell proliferation and increased percentage of cells in S/G2 cell-cycle phase, which was mediated by the increased expression of *CDK6* (ref. ^24^) and *CCL1*,^25^ in contrast to cell-cycle arrest in G0/G1 phases following GAS5 ectopic expression.^24,25,27^ Additionally, overexpression of GAS5 was highlighted to stimulate apoptotic cell death and to reduce cell viability by recruiting E2F4 to *EZH2* promoter to repress gene expression,^26^ as well as to promote doxorubicin-induced apoptosis through depressed expression of *BCL2* (ref. ^27^) in BlCa cells.

Taking advantage of GAS5 powerful and independent prognostic value for NMIBC, we have evaluated the ability of GAS5 loss to improve the prognostic performance of established disease markers. Indeed, the integration of GAS5 loss resulted in superior positive prediction of disease relapse and progression within Ta/T1LG, intermediate/high EORTC-risk, and negative FFC patients’ groups, and thus in improved risk-stratification specificity. In this regard, decision curve analysis highlighted the superior clinical benefit of the prediction model incorporating GAS5 loss for NMIBC relapse and progression compared to the model of the established and clinically used prognostic markers alone.

Overall, our findings clearly support the use of a NMIBC prognosis prediction model, based on GAS5 expression and the independent clinicalpathologic prognostic markers of NMIBC, namely tumour stage, tumour grade and EORTC-risk stratification, using biopsy specimens of TURBT-treated TaT1 patients. This will benefit the identification of the NMIBC patients with higher risk for disease relapse and progression to invasive disease stages, and thus suitable for early curative RC (mainly for T1 patients) or advanced adjuvant treatment with BCG for high-risk Ta patients. Moreover, this prognosis prediction model could be of assistance in the adjustment of NMIBC patients’ post-treatment monitoring to reflect the individual patient’s degree of risk. Similarly, a number of novel molecular prognostic markers for NMIBC have been recently documented; thus, large-scale clinical validation studies, based on independent institutional cohorts, will indicate the best-suited multiplex prediction model/algorithm, incorporating both clinicopathological and molecular disease markers.

In conclusion, GAS5 tumour-suppressor lncRNA is significantly downregulated in bladder urothelial carcinoma, to the extent that it is able discriminate bladder tumours from the normal bladder urothelium. GAS5 loss was correlated with unfavourable disease features, such as invasive disease stages and HG tumours, as well as high EORTC-risk group and positive FFC of the NMIBC (TaT1) patients. Considering disease outcome, GAS5 loss was strongly associated with higher risk for NMIBC early relapse and progression to invasive disease stages following tumour resection, independently of tumour stage, grade, EORTC-risk score and patient’s age and gender. MIBC survival analysis did not revealed a statistically strong association of GAS5 with patients’ survival outcome, indicating the lower impact of GAS5 loss in the biology and the clinical behaviour of muscle-invasive tumours, and supporting the NMIBC-specific prognostic value of GAS5. Hedegaard et al. (*n* = 476) and TCGA provisional (*n* = 413) validation cohorts clearly confirmed the association of GAS5 loss with invasive and HG tumours of non-papillary histology, as well as with NMIBC adverse disease outcome compared to patients overexpressing GAS5. Finally, prediction models incorporating GAS5 loss resulted in superior stratification specificity and improved positive prediction of NMIBC patients’ poor survival outcome following tumour resection, offering a significantly higher clinical benefit for patients’ prognosis and monitoring compared to models of the established and clinically used prognostic markers alone.

## Electronic supplementary material

Supplementary Table 1

Supplementary Table 2

Supplementary Table 3

Supplementary Figure 1
