# Supplementary material for: Loss of GAS5 tumour suppressor lncRNA: an independent molecular cancer biomarker for short-term relapse and progression in bladder cancer patients
Source: Br J Cancer. 2018 Oct 30;119(12):1477–86. doi: 10.1038/s41416-018-0320-6 (PMC6288135; doi:10.1038/s41416-018-0320-6)
Supplement: Supplementary file 1 — Supplementary Table 1 [file 41416_2018_320_MOESM1_ESM.docx]

**Supplementary Table 1. Clinicopathological features of the screening cohort**

| **Variable** | **No. of patients**  **n=176** |
| --- | --- |
| **Disease**  NMIBC (Ta, T1)  MIBC (T2-T4) | **119** (67.6%)  **57** (32.4%) |
| **Tumor stage**  pTa  pT1  pT2  pT3  pT4 | **61** (34.7%)  **58** (33.0%)  **26** (14.8%)  **17** (9.7%)  **14** (8.0%) |
| **Grade (WHO 2004)**  Low  High | **75** (42.6%)  **101** (57.4%) |
| **Grade (WHO 1973)**  1  2  3 | **17** (9.7%)  **66** (37.5%)  **93** (52.8%) |
| **Gender**  Male  Female | **147** (83.5%)  **29** (16.5%) |
| **Non-muscle invasive bladder cancer (NMIBC; TaT1)** | |
| **EORTC risk group**  Low risk  Intermediate risk  High risk | **15** (12.6%)  **38** (31.9%)  **66** (55.5%) |
| **Disease monitoring**  Follow-up patients  Recurrence / Progression  Event-free survival  Excluded from follow-up | **102**  **40** (39.2%) / **15** (14.7%)  **62** (60.8%)  **17** |
| **Muscle-invasive bladder cancer (MIBC; T2-T4)** | |
| **Disease monitoring**  Follow-up patients  Alive  Death  Excluded from follow-up | **49**  **24** (49.0%)  **25** (51.0%)  **8** |
