# Supplementary material for: Loss of GAS5 tumour suppressor lncRNA: an independent molecular cancer biomarker for short-term relapse and progression in bladder cancer patients
Source: Br J Cancer. 2018 Oct 30;119(12):1477–86. doi: 10.1038/s41416-018-0320-6 (PMC6288135; doi:10.1038/s41416-018-0320-6)
Supplement: Supplementary file 2 — Supplementary Table 2 [file 41416_2018_320_MOESM2_ESM.docx]

**Supplementary Table 2. Logistic regression analysis for the discrimination of bladder tumors from matched adjacent normal bladder tissues according to GAS5 levels**

|  | **Bladder tumors *vs* matched adjacent normal bladder tissues** | | |
| --- | --- | --- | --- |
|  | ***Univariate Analysis*** | | |
| Covariant | **OR^a^** | **95% CI^b^** | ***p*-value^c^** |
| **log_10_GAS5** | 0.332 | 0.184-0.599 | <0.001 |

^a^ Odds ratio

^b^ Confidence interval of the estimated OR

^c^ Test for trend
