# Supplementary material for: Loss of GAS5 tumour suppressor lncRNA: an independent molecular cancer biomarker for short-term relapse and progression in bladder cancer patients
Source: Br J Cancer. 2018 Oct 30;119(12):1477–86. doi: 10.1038/s41416-018-0320-6 (PMC6288135; doi:10.1038/s41416-018-0320-6)
Supplement: Supplementary file 3 — Supplementary Table 3 [file 41416_2018_320_MOESM3_ESM.docx]

**Supplementary Table 3. Cox regression analysis for the prediction of NMIBC (TaT1) patients risk for relapse (DFS) and progression to invasive tumors (PFS) following TURBT according to GAS5 levels.**

|  | ***Univariate analysis*** | | | | | | | | | | | | | |
| --- | --- | --- | --- | --- | --- | --- | --- | --- | --- | --- | --- | --- | --- | --- |
|  | **Disease-free survival (DFS)** | | | | | | | **Progression-free survival (PFS)** | | | | | | |
| **Covariant** | **HR^a^** | **95% CI^b^** | ***p*-value^c^** | **Bootstrap**  **BCa 95% CI^d^** | | **Bootstrap**  ***p*-value^c^** | | **HR^a^** | | **95% CI^b^** | ***p*-value^c^** | **Bootstrap**  **BCa 95% CI^d^** | **Bootstrap**  ***p*-value^c^** | |
| **GAS5**  High expression  Low expression | 1.00  2.659 | 1.348-5.246 | 0.005 | 1.439-5.847 | | 0.003 | | 1.00  6.628 | | 1.494-29.403 | 0.013 | 1.804-92.944 | 0.005 | |
| **Tumor Stage**  Ta  T1 | 1.00  1.664 | 0.930-2.976 | 0.086 | 0.954-3.019 | | 0.064 | | 1.00  4.501 | | 1.481-13.682 | 0.008 | 1.568-26.629 | 0.005 | |
| **Tumor Grade**  Low  High | 1.00  1.693 | 0.943-3.040 | 0.078 | 0.919-3.007 | | 0.077 | | 1.00  4.113 | | 1.542-10.971 | 0.005 | 1.480-12.516 | 0.003 | |
| **EORTC risk group**  Low *vs* Int. *vs* High | 1.350 | 0.875-2.082 | 0.175 | 0.900-2.257 | | 0.159 | | 2.486 | | 1.018-6.075 | 0.046 | 0.929-30.938 | 0.059 | |
| **Gender**  Male  Female | 1.00  1.661 | 0.800-3.450 | 0.173 | 0.686-3.618 | | 0.153 | | 1.00  1.910 | | 0.628-5.806 | 0.254 | 0.362-4.948 | 0.242 | |
| **Age** | 0.994 | 0.965-1.025 | 0.716 | 0.969-1.021 | | 0.693 | | 1.019 | | 0.970-1.071 | 0.457 | 0.966-1.083 | 0.444 | |
|  | ***Multivariate analysis^e^*** | | | | | | | | | | | | | |
|  | **Disease-free survival (DFS)** | | | | | | **Progression-free survival (PFSS)** | | | | | | | |
| **Covariant** | **HR^a^** | **95% CI^b^** | ***p*-value^c^** | **Bootstrap**  **BCa 95% CI^d^** | **Bootstrap**  ***p*-value^c^** | | **HR^a^** | | **95% CI^b^** | | ***p*-value^c^** | **Bootstrap**  **BCa 95% CI^d^** | | **Bootstrap**  ***p*-value^c^** |
| **GAS5**  High expression  Low expression | 1.00  2.680 | 1.248-5.753 | 0.011 | 1.270-7.078 | 0.011 | | 1.00  6.362 | | 1.144-35.388 | | 0.035 | 0.705-1.197x10^9^ | | 0.041 |
| **Tumor Stage**  Ta  T1 | 1.00  1.317 | 0.426-4.075 | 0.632 | 0.369-5.270 | 0.615 | | 1.00  32.367 | | 0.526-1.991x10^3^ | | 0.098 | 0.536-3.952x10^10^ | | 0.054 |
| **Tumor Grade**  Low  High | 1.00  0.856 | 0.362-2.028 | 0.724 | 0.360-1.941 | 0.697 | | 1.00  2.509 | | 0.511-12.316 | | 0.257 | 0.417-8.480x10^4^ | | 0.240 |
| **EORTC risk group**  Low *vs* Int. *vs* High | 0.948 | 0.413-2.179 | 0.901 | 0.406-2.389 | 0.897 | | 0.119 | | 0.012-1.185 | | 0.070 | 1.223x10^-5^-0.829 | | 0.022 |
| **Gender**  Male  Female | 1.00 2.039 | 0.875-4.753 | 0.099 | 0.796-5.129 | 0.066 | | 1.00 4.564 | | 0.960-21.704 | | 0.056 | 5.257x10^-6^-129.801 | | 0.043 |
| **Age** | 0.997 | 0.965-1.030 | 0.858 | 0.961-1.033 | 0.857 | | 1.009 | | 0.948-1.074 | | 0.772 | 0.891-1.254 | | 0.813 |

^a^ Hazard Ratio

^b^ 95% confidence interval of the estimated HR

^c^ calculated by test for trend. Bootstrap *p*-value is based on 1000 bootstrap samples

^d^ Bootstrap bias-corrected and accelerated 95% confidence interval of the estimated HR based on 1000 bootstrap samples

^e^ Multivariate analysis adjusted for GAS5 levels, tumors’ stage, tumors’ grade, EORTC-risk group stratification, patients’ gender and age
