# Supplementary figures and images for: Loss of GAS5 tumour suppressor lncRNA: an independent molecular cancer biomarker for short-term relapse and progression in bladder cancer patients
Source: Br J Cancer. 2018 Oct 30;119(12):1477–86. doi: 10.1038/s41416-018-0320-6 (PMC6288135; doi:10.1038/s41416-018-0320-6)

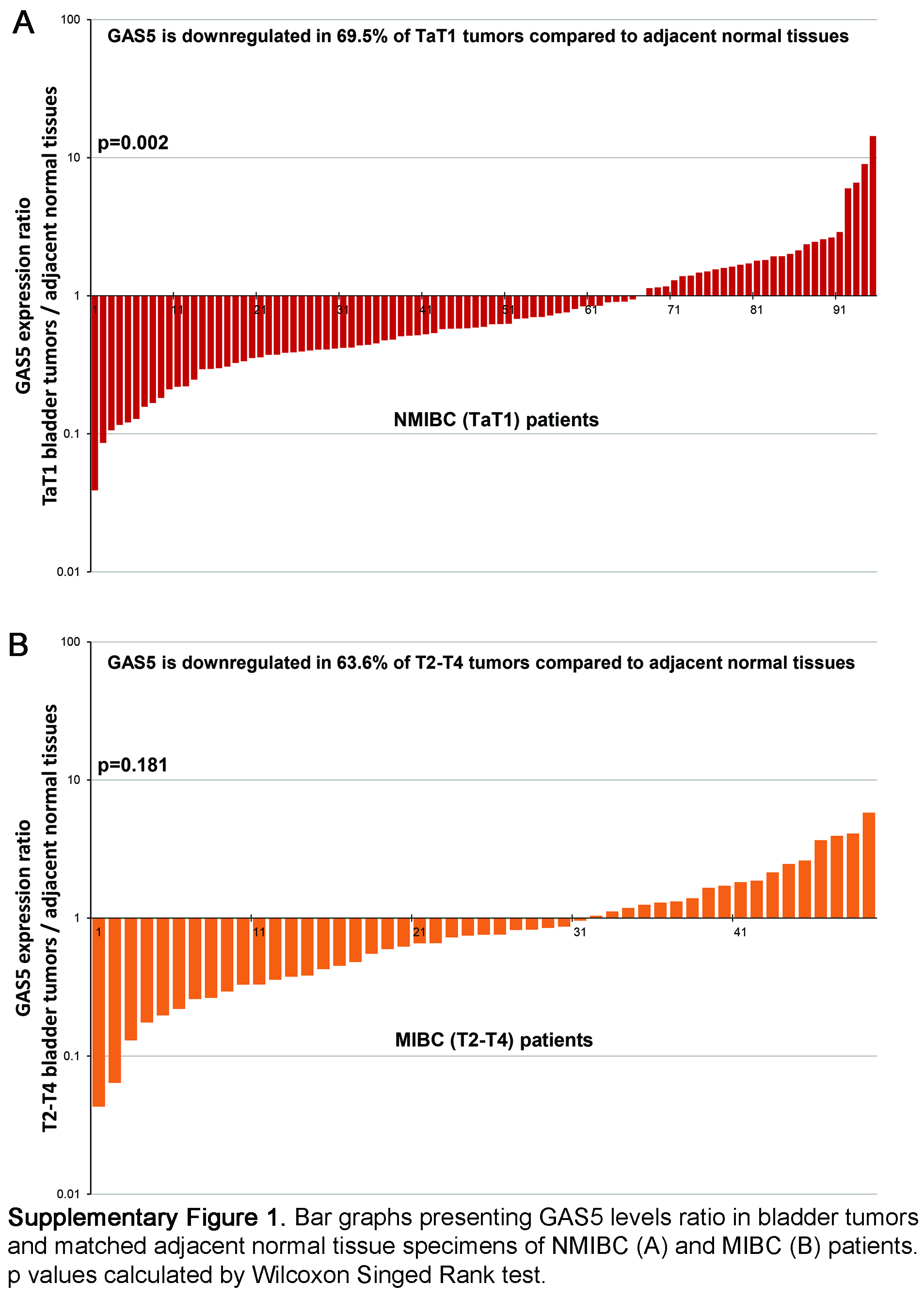

Supplement: Supplementary file 4 — Supplementary Figure 1 [file 41416_2018_320_MOESM4_ESM.tif]
